# Progress in State Medicine

**Published:** 1902-04-05

**Authors:** 


### Progress in State Medicine.

### SMALL-POX.

Blyth[1] discussing the prevention of small-pox in the Metropolis draws attention to the fact that since 1871 there has been very little of that disease in this country. One attack of an infectious fever, such as small-pox, measles, and scarlet fever rendered the person less liable to contract a second, particularly if the second infection followed close after the first. Nevertheless second and even third attacks of the same infectious fever occasionally occurred. This depended on the individual, and bore some relation to the time which had elapsed since the first attack; immunity had a tendency to wear out. It was absurd to expect a life-long immunity from vaccination in childhood. The crude statistics relating to small-pox and vaccination were misleading, because the public understood by the word "vaccinated" protected or immunised. They require to be more carefully tested and classified in order to arrive at their true interpretation. In the preliminary statistics published by the Metropolitan Asylums Board the 1,017 cases were divided into two groups, 843 vaccinated and 194 unvaccinated. The vaccinated group showed that owing to the protection of vaccine, lasting from five to fifteen years, the main incidence was on adults, least incidence on children, and the effect of primary vaccination protecting children was evidenced in the many epidemics during the last century. The protective influence of revaccination was illustrated by the crowd of revaccinated nurses who walked in an atmosphere of infection unharmed, unattacked; in a partially protected community, on the other hand, the slightest chance contact bore fruit. Vaccination should be very recent and very thorough to absolutely protect. The spread of small-pox was without doubt assisted by certain insanitary conditions, but stopped up closets, sewer and drain gas, filthy collections of matter, bad smells generally, and noisome habitations in no way affected the spread of the malady. No connection had been established between any of these things in any epidemic. The only insanitary condition which had a marked influence on small-pox was overcrowding and especially night overcrowding. The one single condition which predominated over all sanitary conditions was that of immunity, whether acquired from a previous attack of small-pox or from efficient vaccination. Out of the local hospital staff, numbering 40 persons, during the Leicester epidemic, 34 were more or less immune either from revaccination or a previous attack of small-pox. One of those protected persons, whose revaccination was ten years old, suffered from a mild attack. Of the remaining six who refused vaccination, five contracted small-pox, and one died. Careful study of 50 years of deaths, and 15 years of well-kept records of admission into hospital indicated that the present epidemic, if it followed the course of previous outbreaks, would increase up to May, and not decline until the first or second week in June. In previous epidemics there had been a fall in February or March, and with the commencement of April a marked outburst. The most potent preventive is vaccination and revaccination. A London citizen who did not in this way protect himself and his household failed in his duty. Such neglect was criminal. Treatment by isolation in hospital is indispensable. The homeless class constitute a danger, there being no possibility of keeping them under observation. Would that there existed a Minister of Health, clothed with despotic power, and with the courage to exercise it, who would issue a mandate for the immediate compulsory vaccination of all in health who bore no evidence of recent vaccination; the scourge would be stayed, and there would be very little small-pox in the metropolis for years to come. In the interim report [2] of the Statistical Committee of the Metropolitan Asylums Board, it is stated that towards the end of January, 1901, five cases were admitted into the Board's hospitals, one in the middle of February, but during March, April, and up to May 29th not a single case occurred. On the last-mentioned date a patient was admitted from Islington, on June 10th one from Whitechapel, on June 29th one from Hackney, and on June 28th and 29th one on each day from Wandsworth. During the month of July and up to August 21st single cases occurred in various parts of London. Subsequently cases occurred in every one of the 31 Poor Law parishes and unions comprising the Metropolitan Asylums District. The gross mortality is given as 24·29 per cent., but this is undoubtedly higher than it will be when all the cases admitted during 1901 have been completed. With reference to vaccination the cases were divided into three classes: (1) "Vaccinated"—that is, cases having visible cicatrices; (2) "doubtful," including (*a*) cases stated to have been vaccinated, but bearing no visible evidence thereof, and (*b*) cases in which no statement was made, but in which the eruption prevented any reliable observation as to cicatrices; and (3) "unvaccinated," that is cases admittedly unvaccinated, or bearing no marks of the operation, and as to which no statement was made. With regard to age the committee point out that under 10 there were only 12 vaccinated cases and no death; six doubtful cases, all of whom died; and 95 unvaccinated cases, of whom 52 died—a percentage of 54·74. Under 20 years of age there were 161 vaccinated cases, of whom three died, a percentage of 1·86; 12 doubtful cases, of whom seven died, a percentage of 58·33; and 161 unvaccinated cases, of whom 79 died, a percentage of 49·07. They also point out that there appears to be a distinct diminution in the protective power afforded by primary vaccination after the age of 20 years; the death-rate rising from 9·85 in vaccinated cases between 20 and 25 years of age, to 28·95 in cases between 35 and 40. Among the patients admitted during the year there were no fewer than 21 who were employed on disinfecting work (of whom one died), most of them being servants of borough councils. Not one of these persons had been revaccinated since infancy. Against this, attention is drawn to the experience of the managers at their small-pox hospitals and in the ambulance service for many years past, in both of which services revaccination is always performed on engagement. Of 2,198 persons employed at the small-pox hospitals between 1884 and 1900 inclusive, in which period 17,900 small-pox cases were received into the hospitals, only 17 persons contracted small-pox, of whom 13 were not revaccinated until after they had joined the ship, and four were workmen who escaped medical observation. Not one of the staff of the hospital ships has ever died of small-pox, and not one has ever even suffered from the disease for the past eight years. From the year 1881 to the end of 1901 there have been employed in the ambulance service of the board 1,282 persons. Four of these contracted small-pox, of whom one escaped vaccination when appointed—he died; one was unsuccessfully revaccinated on joining the service, and the operation was not repeated—she died; the two others had been revaccinated and recovered. The prevalence [3] of small-pox in Essex is rapidly increasing, 500 cases being notified during August 1901, to February 8, 1902, of which no fewer than 319 have occurred in the Orsett Union having a population of 33,721. The cases per 1,000 persons up to the end of January in the Orsett Union were 8·60; for the remainder of the county 0·21. The small-pox ships of the Metropolitan Asylums Board lie near the Kent shore opposite to the hamlet of Purfleet, in West Thurrock parish in the Orsett Union. The ships are about 700 yards from the shore, and the whole of the hamlet of Purfleet lies within a radius of three-quarters of a mile from the ships as a centre. The County Medical Officer of Health gave it as his opinion that the epidemic was caused by airborne infection from the ships, whilst the Metropolitan Asylums Board suggested that it was due to personal communication between the ships and the shore. For several years now there has been no such communication, and yet as soon as a number of cases of small-pox were received on the ships an outbreak occurred in Purfleet. Nearly four times as many cases have occurred in Purfleet as in the more distant portion of West Thurrock, and fourteen times as many as in the rest of the Orsett Union. No cause for this excessive prevalence can be found save the proximity of the hospital ships. Further evidence adduced in favour of the air-borne theory is based on the direction of the wind during the past six months. For the greater part the wind has merely veered from west to south-west; on a very few days has the wind had a northerly direction. One portion of Purfleet, the smaller, lies almost due north of the ships, the other almost due east. The former has only on a few occasions been exposed to wind blowing from the ships, and here the case per cent. of population was 0·8; whilst the latter has been almost constantly exposed to such winds, and here the case per cent. of the population was 12·0.

The Essex County Council suggests either that the use of the ships be at once entirely abandoned, or that they should be used for convalescent cases only. Hope[4] in a short report on the Vaccination Act of 1898, states that one of the most important requirements in an amended Act should be the transference of the control of vaccination from boards of guardians to sanitary authorities. If this were done revaccination would be facilitated and more systematically carried out, while the erroneous idea that vaccination is a poor-law rather than a public health measure would be discountenanced. Revaccination should be made compulsory amongst insufficiently protected persons in all houses or establishments where a case of small-pox has occurred, and among all persons employed in or about small-pox hospitals, disinfecting stations, ambulances, etc. The question should be considered whether Government stations for the preparation of vaccine should not be established in some large provincial centres. Finally, he doubts whether the conscience clause has been a success, and is of opinion that the result has not been to "conduce to increased vaccination." Harris[5] draws attention to the difficulty in some cases of procuring information in order to trace the source, or prevent the spread of infection. He strongly recommends that the matter be brought to the knowledge of the Local Government Board and the County Council, with a view to an enactment being passed, making it a punishable offence to refuse or withhold information, or to give misleading information respecting the number of persons residing in an infected house, or their names, employment or occupation, or the names of their employers, or the schools attended by them, or as to any trade, business, employment, or manufacture that may be conducted on the premises.

[1] Pub. Health Engineer, Feb. 22, 1902. [2] Brit. Med. Jour., Jan. 18, 1902. [3] Brit. Med. Jour., Feb. 15, 1902. [4] Brit. Med. Jour., Mar. 8, 1902. [5] Sanit. Record, Dec. 26 1901.

